# Knowledge and Perceptions of Non-Nutritive Sweeteners Within the UK Adult Population

**DOI:** 10.3390/nu13020444

**Published:** 2021-01-29

**Authors:** Grace Farhat, Fleur Dewison, Leo Stevenson

**Affiliations:** School of Health Sciences, Liverpool Hope University, Hope Park, Liverpool L16 9JD, UK; 16004159@hope.ac.uk (F.D.); Stevenl2@hope.ac.uk (L.S.)

**Keywords:** non-nutritive sweeteners, low-calorie sweeteners, consumer perception, food safety, weight control, diabetes, cancer risk

## Abstract

Non-nutritive sweeteners (NNS) are popular sugar substitutes that can help in weight and diabetes management, but concerns regarding their use have been raised by the public. This study aimed to investigate knowledge, benefits and safety perceptions of NNS in a sample of UK adults. The impact of knowledge dissemination on the change in perceptions was also examined. An online survey was distributed through social media platforms and UK Universities and was completed by 1589 participants aged 18 years and above. Results showed a high-risk perception of NNS and a lack of knowledge in regulations in nearly half the population sample. The artificial attributes of NNS further limited their acceptance. Risk perception has been significantly linked to a lower consumption of sweeteners (*p* < 0.001) and was affected by gender, occupation, education levels, age and body weight status. Information dissemination significantly reduced risk perception and increased awareness of the benefits of NNS. Results suggest that developing effective communication strategies to educate consumers, potentially through trusted health government agencies and professional bodies, can help them to make informed choices. Education of health professionals could also be valuable in reassuring the public of the benefits of NNS.

## 1. Introduction

Non-nutritive sweeteners (NNS), also referred to as low-calorie sweeteners (LCS) or artificial sweeteners, are popular sugar substitutes, providing strong sweetening effects without adding sugar and energy to the diet [1]. The most popular NNS include aspartame, saccharin, sucralose, stevia and acesulfame K [1]. NNS possess different chemical structures and metabolic effects but are comparable in their ability to activate taste receptors [2,3].

NNS have been increasingly consumed to lower energy intake [2] and therefore reduce obesity and diabetes risk. They have, however, been paradoxically involved in weight gain and risk of Type 2 diabetes [2]. A substantial body of evidence disclaimed these effects, reporting a beneficial role of NNS in controlling energy intake and reducing appetite, with potential favourable effects on glucose homeostasis. These effects have been evidently described in expert consensus statements on LCS [4,5]. There has also been a significant public distrust in the safety of NNS. The reported link between saccharin, aspartame and sucralose and cancer risk in animals [6,7,8,9] has cautioned the public against the use of NNS, despite these studies being subsequently discredited, and partly attributing the outcomes to mechanisms in animals that are not applicable to humans [10]. Extensive safety evaluation corroborated the safety of NNS [11], and several organisations such as the American Heart Association (AHA), the American Diabetes Association (ADA) and the British Dietetic Association (BDA) issued statements advocating their safety [12,13].

Even with professional body endorsement worldwide, consumers remain sceptical of the safety of artificial sweeteners [13,14]. A US survey showed that 64% of the population is concerned about the safety of NNS, as reported by Gardner et al. [13]. These concerns could be due to miscommunication of information, or a lack of knowledge in the benefits and risks surrounding the use of NNS; the latter has been described as a barrier to acceptance [15]. Consumers’ knowledge and trust in regulations have also been reported to affect benefits and risk perceptions of sweeteners [16]. Furthermore, “naturalness” has been deemed crucial in consumer approval of foods [17,18]; the term “artificial sweeteners”, which often extends to include stevia [19,20], has been negatively perceived by consumers [13]. This suggests that consumer education might help to promote appropriate messages and avoid misleading information.

Perceptions and trends in NNS consumption vary between countries; therefore, understanding the factors affecting these trends and perceptions can help to develop effective communication strategies for educating the public. The expert consensus on LCS identified a gap in knowledge in relation to the factors influencing consumer perceptions [4]. This survey aimed to collect quantitative and qualitative data and assess benefits and safety perceptions of NNS in a sample of UK adults. The impact of knowledge, trust in regulations and sociodemographic factors was examined. We also aimed to assess whether providing people with information from regulatory authorities and professional bodies can help to change their perceptions of NNS. Outcomes will help to develop approaches for risk communication that can help people to make informed choices.

## 2. Materials and Methods

An online survey (www.onlinesurveys.co.uk) was made available between June 2020 and January 2021 and promoted via multiple social media platforms (Facebook, Twitter) for convenience sampling and for the purpose of accessing a diverse population of online users living in different parts of the country, and with different educational backgrounds and professions. The survey was also promoted in universities for convenience sampling. We used pre-validated statements from a previous study by Bearth et al. [16] who developed a questionnaire based on a preliminary qualitative study and looked at consumers’ perceptions of additives (including artificial sweeteners), trust in regulatory bodies and knowledge in regulations. Adult (18+) UK residents with internet literacy were eligible to take part. Participants were provided with an online information sheet before consenting online to taking part in the survey. No identifiable information (such as name, date of birth) was retained. Ethical approval was granted by the Liverpool Hope University School Ethics Committee. We primarily used the term artificial sweeteners to describe NNS in this survey, since it is most commonly used in the media and professional organisations. However, the other definitions used interchangeably (e.g., non-nutritive sweeteners, low-calories sweeteners) were clearly stated in the survey. The survey is available in Supplementary Survey S1.

### 2.1. Data Collection

Sociodemographic characteristics (age, gender, ethnicity, education, profession, residence) and information about body weight perception and disease history were collected. Participants were asked to rate statements relating to their usual consumption of NNS, main reasons for consumption, risk and benefit perceptions, as well as knowledge and trust in regulations using a 5-point Likert scale (strongly disagree, disagree, neither agree nor disagree, agree, strongly agree). Qualitative data concerning NNS-containing foods and drinks and perceived differences between different types of sweeteners were collected. The second stage of the survey involved sharing positions of professional and regulatory bodies in relation to the benefits and safety of NNS. Respondents were provided with a text stating the position of the BDA and European Food Safety Authority (EFSA) in relation to the beneficial role of NNS in weight management and glucose homeostasis, and their safety concerning cancer risk and other threats to health. They were then asked to immediately rate risk and benefit perceptions again (Supplementary Table S1). 

### 2.2. Sample Size Calculation and Statistical Analysis

We estimated that a minimum sample size of 1037 participants was required for this survey, assuming that the population size of UK adults is 53 million and considering a 4% error margin, 99% confidence interval and 50% response distribution.

Data were analysed using SPSS (27.0 Chicago, IL, USA). Frequencies were used to present population characteristics and main study results. Internal consistency of the survey was assessed using Cronbach’s alpha coefficient. The association between survey outcomes and dependent variables (age, education, profession, gender, weight status and trust in regulations) was examined using ordinal logistic regression. The relationship between body weight status and consumption of sweeteners was assessed using Pearson’s Chi square. Differences in benefits and safety perceptions after knowledge dissemination were assessed using Wilcoxon test. Significance was set at *p* ≤ 0.05.

## 3. Results

A total of 1589 participants completed the survey. Females were overrepresented in this population (84%), and participants in the age range of 45–54 years made up the largest number of respondents (27.8%). Graduates represented 56.4% of the population. Around half the population reported being overweight (48%) and 16% being obese, while 34.2% described themselves as normal weight. Additionally, 66.5% of the population stated being currently or previously on a weight loss diet. Sociodemographic and health characteristics of this population are presented in Table 1. Cronbach’s alpha coefficient showed a good internal consistency for benefits (*α* = 0.86), safety (*α* = 0.91) and trust and knowledge of regulations (*α* = 0.73). 

### 3.1. Source of NNS in Foods and Drinks

**Figure 1:**
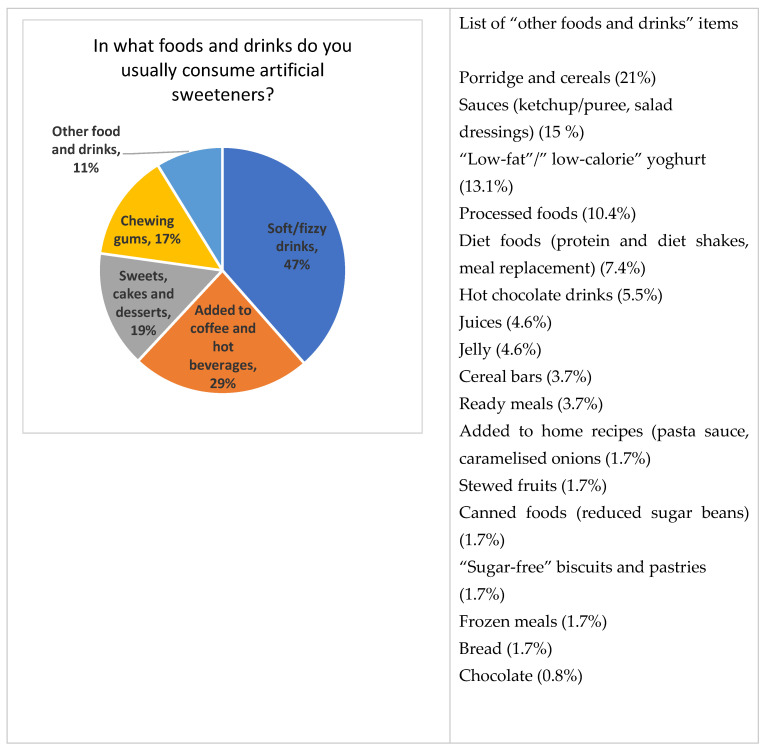
Food and drink sources of non-nutritive sweeteners (NNS) in the survey sample. Values represent percentages of responses to a multi-choice question.

NNS was reported to be consumed by 61.8% of the population, while 38.2% indicated no consumption at all. Pearson’s Chi square showed a significant association between weight status and NNS consumption (*χ*(3) = 44.11, *p* < 0.001), with 67% of overweight and obese consuming NNS, compared to 51% among those who are normal weight. The self-reported food and drink sources of NNS are presented in Figure 1, with beverages and table-top sweeteners constituting the most common source. The most popular brands used are reported in Supplementary Table S1.

### 3.2. Reasons for Consuming NNS

Participants rated statements relating to the reasons for NNS consumption on a 1 to 5 Likert scale, and results are displayed in Table 2. The low energy content of sweeteners is a primary reason for the consumption, alongside the common availability of these substances in many food products. However, 25.3% of respondents stated that, to their knowledge, they do not consume foods and drinks containing NNS, and 38.3% indicated that they check food labels for the presence of artificial sweeteners. 

### 3.3. Knowledge and Perceptions of Safety and Benefits of NNS

**Figure 2:**
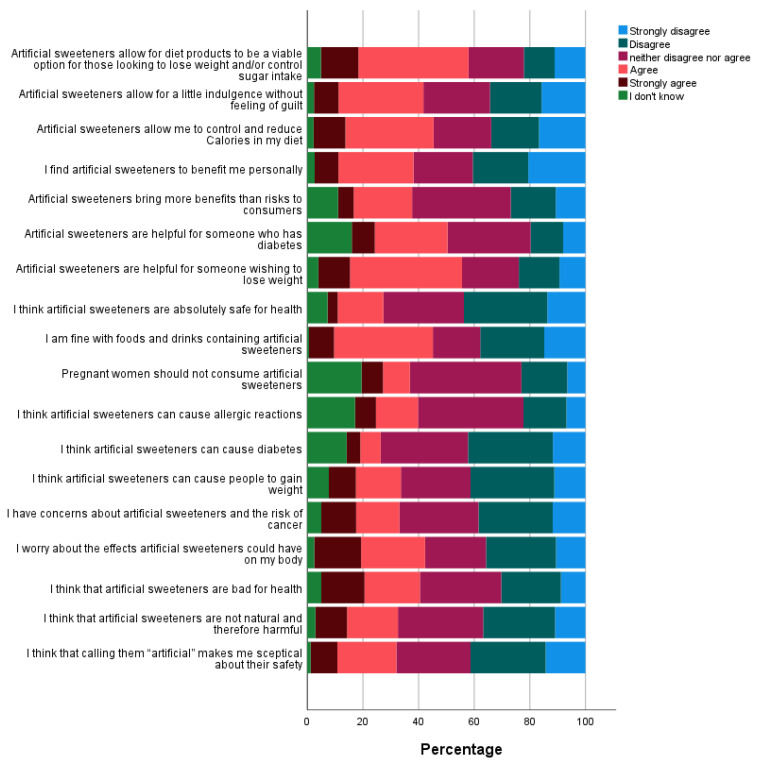
Benefits and safety perceptions of NNS.

Participants rated statements relating to their perceptions of benefits and risks of artificial sweeteners. Responses were spread throughout the 5 levels of the Likert scale (Figure 2). As seen in Figure 2, a considerable percentage of participants identified NNS as being harmful and were concerned about their risks. Regression analysis showed an association between perceived risk and consumption of NNS; those who do not consume sweeteners identified them as worse for health (0.37 (95% CI 0.26 to 0.05) Wald *χ*^2^(1) = 347, *p* < 0.001). Those in the overweight and obese category were more in agreement that NNS benefited them personally compared to those who reported to be normal weight (*p* = 0.04), yet weight status did not affect perceptions of safety and risks (*p* > 0.05). 

#### 3.3.1. Influence of Age on Perceptions

Compared to older age categories, participants aged less 35 years were less in agreement with NNS being not natural and harmful (*p* < 0.05). Those in the age category 25–34 years disagreed more with the statement that artificial sweeteners are bad for health (*p* = 0.048), and they worried less about their carcinogenic effects (*p* = 0.04) compared to older age categories. They also agreed more that NNS can help people to lose weight (1.83 (95% CI (1.21 to 2.78) Wald *χ*^2^(1) = 8.13, *p* = 0.004) compared to older respondents.

#### 3.3.2. Influence of Gender on Perceptions

Women have been reported to be more worried about the effects of artificial sweeteners than men (*p* < 0.001). They regarded them as more harmful (*p* < 0.001), and they worried more about the associated cancer risk (*p* = 0.01), diabetes risk (*p* < 0.001) and weight gain (1.62 (*p* < 0.001)). It is, however, worth mentioning that males constituted only 16% of the survey population.

#### 3.3.3. Influence of Profession on Perceptions

Although health professionals disagreed more with the statement that artificial sweeteners are bad for health (0.62 (85% CI (0.44 to 0.89) Wald *χ*^2^(1) = 6.61, *p* = 0.01) and were less worried about their effects (*p* < 0.05), they did not differ in their views towards their benefits as well as their role in weight gain and diabetes risk when compared to non-health professionals (*p* > 0.05). Those in sales and customer services were more in agreement than other professions that NNS help to reduce calories in the diet (*p* = 0.01).

#### 3.3.4. Influence of Education Level on Perceptions

Although participants with no formal qualifications were more in agreement that artificial sweeteners are not natural and therefore harmful (0.43 (85% CI (0.239 to 0.79) Wald *χ*^2^(1) = 7.459, *p* < 0.001)), they were, in contrast, less in agreement that sweeteners are bad for health (0.42 (85% CI (0.23 to 0.76) Wald *χ*^2^(1) = 8.32, *p* < 0.001)), and they worried less about their risks compared than those with higher education levels (*p* = 0.03). They also agreed more that artificial sweeteners are safe to consume (*p* = 0.01). No other differences in perceptions between other levels of education were noted (*p* > 0.05).

### 3.4. Sources of Consumers’ Knowledge of Benefits and Safety of NNS

**Figure 3:**
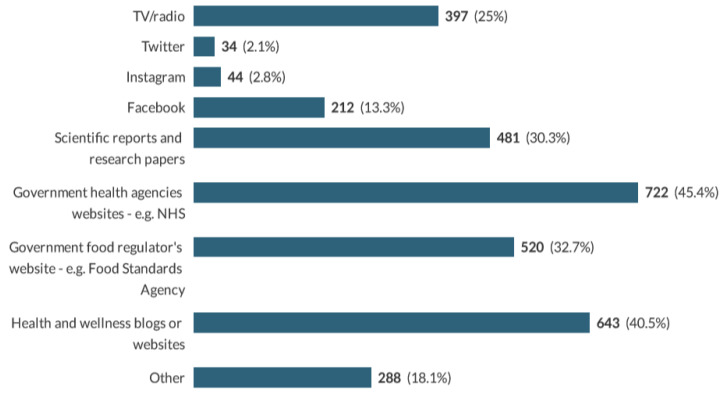
Sources of respondents’ knowledge of benefits and safety of NNS. NHS: National Health service.

Participants were asked to select multiple answers stating their source of knowledge and information about NNS. Results showed that government health agencies and regulatory bodies are the primary source of information, followed by media and wellness blogs (Figure 3). Other sources of information included magazines and newspapers, books, anecdotal articles, diabetes clinics, print media, media outlets, slimming groups, teachers, dietitians, nutritionists, internet browsing, friends, word of mouth and personal views. Among participants who answered “Other”, 26% stated they do not seek to find this information anywhere.

### 3.5. Knowledge and Trust in Regulations Surrounding the Use of Artificial Sweeteners

Knowledge in regulations of NNS was explored, and results are presented in Table 3. It was reported that 42.8% of participants lack awareness in regulations, with 50.3% of participants lacking motivation to look for them. Regression analysis showed a significant association between awareness/knowledge of regulations and perceptions of sweeteners; those who are aware of regulations were more in favour of the benefits of NNS than those who are not (*p* < 0.001). 

Results also showed that the majority of respondents trust regulatory bodies (72.3%), government health agencies (73.7%) and research/scientific papers (77.8%), and 71% distrust information coming from social media. Only 31.9% have doubts about information coming from health and wellness blogs. Nevertheless, trust in regulations did not have an association with the benefits nor safety perceptions of sweeteners (*p* > 0.05). 

### 3.6. Attitudes towards Different Types of Sweeteners

**Figure 4:**
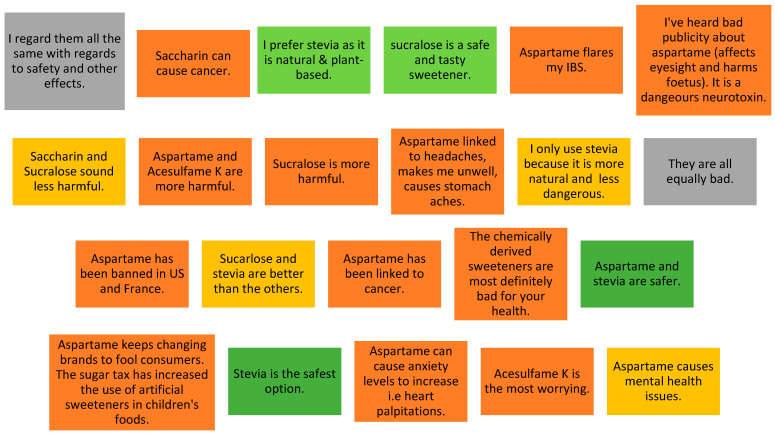
Perceptions towards different types of NNS as reported by survey respondents.

An open-ended question aimed to explore whether participants perceived the types of NNS differently. A combined summary of the most relevant and common answers is displayed in Figure 4. The fact that they are all perceived similarly was a common answer. However, multiple responses indicated a preference for stevia due to its “natural attributes”, but complaints about its bitter taste were raised. Aspartame has been mostly regarded as harmful, with several side effects noted.

The comments of health professionals (*N* = 209) seemed to share similar levels of concern and lack of information as the public, including their opinions of stevia and aspartame. Several comments associated NNS to cancer risk, disruption of gut microbiota, hormonal disturbances and long-term risks. 

### 3.7. Knowledge Dissemination

The second part of the survey aimed to investigate whether sharing information from regulatory and professional bodies will change perceptions of safety and benefits of NNS. After reading the text stating the benefits and safety of NNS, 44% of respondents were not previously aware of this information and 33% stated that they changed their opinions, while 19.4% remained not convinced. Analysis showed that following knowledge dissemination, participants perceived artificial sweeteners to have more benefits than risks (Z = −6.04, *p* < 0.001); they were less worried (Z = −12.88, *p* < 0.001) and less concerned (Z = −8.79, *p* < 0.001). They were also more convinced that NNS do not lead to weight gain (Z = 8.14, *p* < 0.001) or cause diabetes (Z = 2.6, *p* < 0.001) or cancer (Z = 1.96, *p* = 0.05).

Among those worried, 60% stated that they would have been less concerned had they known this information in advance. Participants primarily attributed their concern to the frequent change in medical research, and to a worry of getting accustomed to the sweet taste.

**Figure 5:**
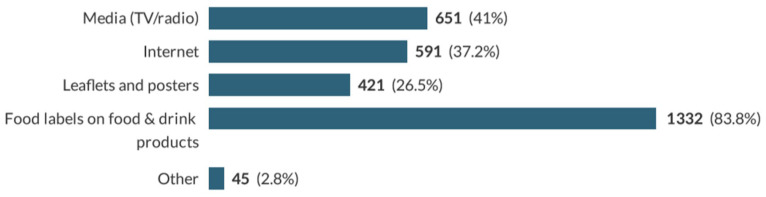
Preferred sources of information for dissemination of safety and benefits of NNS. Other responses included newspapers, schools, and health professionals (doctors, nurses).

Lastly, participants were asked about the best way to communicate effective information to them, and results are displayed in Figure 5. 

## 4. Discussion

This survey intended to investigate the benefits and safety perceptions of NNS in the UK population and look at whether information transmission changed perceptions over a short period of time. The aim was to develop effective communication strategies that help the public to make informed choices. To our knowledge, this is the first UK survey that aimed to look at consumer perceptions of NNS. We have shared valuable information that can be useful for government health agencies, professional bodies, health professionals and food and drink manufacturers and can help to find suitable ways to convey research-based evidence to the public.

The percentage of respondents who reported being overweight and obese is in line with UK figures, indicating that 6 in 10 adults are either overweight or obese [21]. NNS are more commonly consumed in this weight category (67%), presumably to lose weight or avoid further weight gain. This trend has previously explained the association between NNS and obesity in cohort studies, attributing the link to reverse causality [22]. This survey also showed that beverages are the main source of sweeteners, followed by table-top sweeteners, and this agrees with previous studies looking at patterns of consumption in Western society [23,24]. The percentage of graduates in this sample (56.4%) was, nevertheless, higher than the percentage of UK graduates (26.4%) [25]. This might be due to the promotion of the survey in universities and could have led to this population having more access to scientific reports and research papers than the general population. 

NNS are mainly consumed because they have a low energy content (81%), are available in multiple foods and drinks (79.1%) and satisfy food cravings (56.3%). The latter suggests that NNS can have implications in controlling energy intake; it has been suggested in a recent study that in the presence of NNS, participants had lower calorie intake during food cravings [26]. It is not clear, however, whether the use of NNS has led to a lower overall energy intake in our cohort and subsequently prevented obesity in some and further weight gain in others. Only 26.1% of respondents found NNS tasty, suggesting that efforts into improving this attribute are required by food manufacturers. Stevia, for instance, which has been perceived as the healthiest sweetener, has registered complaints due to its bitter taste.

Our study shows that the lack of knowledge in regulations is linked to a lower acceptance of NNS, and consequently a lower consumption. Similar results were reported by a Swiss survey [16]. Nearly half of our population was unaware of the regulations, with some attributing the reason to a lack of motivation to seek relevant information. It is worth noting that a significant percentage of participants used the option “neither agree nor disagree” to answer statements relating to benefit and risk perception. This option has been previously reported to suggest either a lack of knowledge, a dilemma, or a rejection of the statements [27]. These responses alongside those who answered “I don’t know” suggest the need for consumers’ education to help them to make choices based on informed knowledge. 

The artificial attributes of NNS have further lowered their acceptance among this population. Consumer education is therefore essential to dissociate “artificial” from risk. Saraiva et al. (2020) also highlighted that the use of natural sweeteners (as an alternative to synthetic sweeteners) in foods and drinks will increase consumer acceptance and the purchase of these products [18]. This implies that an additional way to increase consumers’ approval is through the use of natural NNS (e.g., stevia) in more low-energy foods and drinks.

This study suggests that simple and easily accessible messages, primarily through media, social media and leaflets could be effective in educating consumers on the benefits of NNS. Our results showed that risk perceptions are higher in women, older adults and those with an education qualification. These findings are consistent with previous studies looking at factors affecting health risk perceptions related to foods [28,29]. It is worth pointing out, however, that participants with no formal qualifications made up only 2.7% of our population, and results involved contradictory statements regarding health perceptions. Therefore, no firm conclusions can be made. As for older age and the associated increase in the prevalence of non-communicable diseases [30], rejecting products with low calorific value due to increased risk perception can exacerbate the issue. Efforts into exploring ways for a better consumer education in practice while taking into account all these factors must then be considered.

Furthermore, adding information about NNS on food packages has been deemed as the preferred suggested method among respondents (83%). In the UK, it is mandatory to display NNS content on food labels [31], yet the practicality of adding more detailed consumer education might raise practical issues about the level of information that can be displayed on a food or drink product.

Trust in government and regulations did not affect perceptions of sweeteners in this survey. However, the high level of trust puts government health agencies in an ultimate position to provide effective communication and reassure consumers about the safety and benefits of NNS. This could be valuable, as by simply sharing information from professional and regulatory bodies, risk perceptions decreased in our population. However, looking at measuring behaviour change beyond this short period of time is needed. A previous Korean study reported promising results, as a month after knowledge transmission, consumer’s perceptions of food additives significantly improved [32]. Regulatory bodies (such as EFSA and FSA) conduct extensive risk assessments and safety evaluations but do not necessarily aim for public communication. Nonetheless, drink manufacturers/retailers would perhaps need to be more mindful of food regulations and regulatory bodies and help to further assure consumers. Professional bodies and health professionals can be very effective in consumer education, yet based on the results of this study, education of health professionals is needed and has been suggested in a previous LCS consensus statement [5]. Health professionals who are particularly involved in the treatment and management of diet-related diseases (such as Type 2 diabetes, obesity, etc.) are best placed to advocate for the health benefits of NNS.

Aspartame has been regarded as a harmful sweetener by no less than 50% of respondents in this study, with several side effects reported. Aspartame has been the subject of internet hoax over the past two decades, which have affected consumers’ views about this sweetener. It is, therefore, important to be aware of this point when developing future communication strategies. Stevia, on the other hand, has been perceived as the safest or the least harmful, particularly due to its natural attribute. 

Based on our survey outcomes, we suggest below a number of recommendations that could help in consumers’ education and awareness:

1. Educate consumers and health professionals that “natural” does not necessarily imply “healthy”. Additionally, as stevia derives from plant sources, we suggest avoiding grouping it under the umbrella of “artificial sweeteners”. We also recommend unifying the labelling of these substances to either NNS or LCS across all online resources of professional organisations and regulatory bodies.
2. Government health agencies (notably the NHS (National Health Service) in the UK) are held in high esteem in this population, and they could therefore promote easy to access information relating to benefits and risks of NNS, particularly through media, social media and leaflets. The feasibility of these initiatives would evidently need to be further explored.
3. The practicality of displaying more information on the benefits of NNS on food packages needs to be investigated.
4. There remains a gap in knowledge in research relating to some health impacts of NNS (e.g., on gut microbiota) [4]. Further studies are essential to provide further evidence of the safety and benefits of NNS for health professionals and the public.

### Strengths and Limitations

This is, to our knowledge, the first UK survey that investigated the benefits and safety perceptions of sweeteners. It included a large population sample and collected both quantitative and qualitative data. Some limitations include the overrepresentation of women and the high education level of this cohort which limit results’ generalisation to the UK population. Additionally, this survey was only distributed online, which restricted participation to internet literates. Furthermore, the study did not inform about the changes in perceptions and/or use of sweeteners beyond the immediate context of the survey. Lastly, while we looked at sources of NNS in foods and drinks, we did not investigate the amounts consumed, nor the perceptions in relation to oral health. 

## 5. Conclusions

This survey shows that there remains a lack of knowledge and a high-risk perception of NNS in this population, despite the trust in government health agencies and regulatory bodies. High-risk perception of NNS affected their acceptance, which has been further limited by their artificial attributes. Effective communication, including simple and clear messages, delivered though media, social media and leaflets, while considering sociodemographic perception-based differences towards sweeteners, can help to disclaim controversial claims and assist consumers in making informed choices. There is also a need to educate health professionals on the benefits and safety of these substances in order to help to promote their benefits to the public.

## Figures and Tables

**Table 1:** Sociodemographic and health characteristics of the survey population.

	Number of Respondents (N = 1589)	Percentage of the Population
**Gender**		
Male	246	15.5%
Female	1339	84.3%
Other	4	0.3%
**Age (years)**		
18–24	110	6.9%
25–34	276	17.4%
35–44	367	23.1%
45–54	441	27.8%
55–64	286	18%
65+	109	6.9%
**Ethnicity**		
White British	1414	89%
White Irish	34	2.1%
Other white	86	5.4%
Mixed race	16	1%
Other	13	0.8%
**Education**		
No formal qualifications	43	2.7%
GCSE/O-level	287	18.1%
A-Level or Equivalent	332	20.9%
Degree level	547	34.4%
Postgraduate level	350	22%
Other	30	1.9%
**Profession**		
Health-related professions	209	12.9%
Managers, directors and senior officials	198	12.5%
Professional occupations (other than health-related)	323	20.3%
Associate professionals or technical	57	3.6%
Administrative and secretarial	165	10.4%
Skilled trade	59	3.7%
Caring, leisure and other service	82	5.2%
Sales and customer service	94	5.9%
Student/unemployed/retired	223	14%
Other	179	11%
**Country of residence**		
England	1309	82.4%
Scotland	57	3.6%
Wales	203	12.8%
Northern Ireland	20	1.3%
**Disease history**		
Type 1 Diabetes	18	1.1%
Type 2 Diabetes	92	5.8%
High blood pressure	208	13.1%
Heart disease	32	2%
Cancer	52	3.3%
None of the above	1266	79.7%

**Table 2:** Reasons for consuming NNS.

I Consume Artificial Sweeteners Because They:	Strongly Disagree	Disagree	Neither Agree nor Disagree	Agree	Strongly Agree
Are tasty	94 (9.6%)	191 (19.5%)	441 (44.9%)	212 (21.6%)	44 (4.5%)
Are healthier than sugars	88 (9%)	180 (18.3%)	255 (26%)	360 (36.7%)	99 (10.1%)
Are low in calories	55 (5.6%)	33 (3.4%)	98 (10%)	393 (40%)	403 (41%)
Satisfy sweet cravings	78 (7.9%)	118 (12%)	236 (24%)	412 (42%)	138 (14.1%)
Are ingredients in foods and products that I consume	52 (5.3%)	36 (3.7%)	117 (11.9%)	447 (45.5%)	330 (33.6%)

Values represent number of respondents (percentage).

**Table 3:** Knowledge of regulations surrounding the use of artificial sweeteners.

Question	Strongly Disagree	Disagree	Neither Agree nor Disagree	Agree	Strongly Agree	I Don’t Know
I am aware of the regulation surrounding the use of artificial sweeteners	203 (12.8%)	632 (39.8%)	295 (18.6%)	303 (19.1%)	47 (3%)	109 (6.6%)
I am not aware of these regulations as I don’t know where to look for them	105 (6.6%)	384 (24.2%)	374 (23.5%)	504 (31.7%)	176 (11.1%)	46 (2.9%)
I am not aware of these regulations as I am not motivated enough to look for them	108 (6.8%)	319 (20.1%)	331 (20.8%)	609 (38.3%)	191 (12%)	31 (2%)
I trust the regulatory bodies as their aim is to protect consumers’ health	67 (4.2%)	172 (10.8%)	360 (22.7%)	765 (48.1%)	186 (11.7%)	39 (2.5%)
I trust the regulator’s position (such as EFSA and FSA) regarding the safety and benefits of artificial sweeteners	61 (3.8%)	155 (9.8%)	362 (22.8%)	784 (49.3%)	171 (10.8%)	56 (3.5%)
Regulations means only a safe amount of these sweeteners are available in foods and drinks	76 (4.8%)	227 (14.3%)	412 (25.9%)	577 (36.3%)	140 (8.8%)	157 (9.9%)
All artificial sweeteners have been vigorously tested before being allowed on the market	96 (6%)	193 (12.1%)	422 (26.6%)	506 (31.8%)	157 (9.9%)	215 (13.5%)

Results are presented as number of respondents (percentage).

## Data Availability

Not applicable.

## Supplementary Materials

The following are available online at https://www.mdpi.com/2072-6643/13/2/444/s1, Survey S1: Consumers’ knowledge and benefit perceptions of artificial sweeteners, Table S1: Brands of NNS-containing foods and drinks commonly used.
