# Supplementary material for: Knowledge and Perceptions of Non-Nutritive Sweeteners Within the UK Adult Population
Source: Nutrients. 2021 Jan 29;13(2):444. doi: 10.3390/nu13020444 (PMC7911612; doi:10.3390/nu13020444)
Supplement: Supplementary file 1 [file nutrients-13-00444-s001.zip › suppl/Supplemental Table S2.docx]

Table S2. Brands of NNS-containing foods and drinks commonly used.

|  | Number of responses |
| --- | --- |
| Pepsi Max/Diet Coca/cola/Diet Pepsi | 349 |
| Sweetex . Hermesetas, Silver spoon | 57 |
| Asda/Robinson’s/Vimto sugar free fruit squash/Jordan’s skinny syrup | 52 |
| Tesco Pink lemonade/other supermarket diet lemonade brands | 43 |
| Robinson's juice/Ribena/Vimto diet | 38 |
| Canderel sweetener | 36 |
| 7up free/Fanta | 35 |
| Orbit/ Wrigley's chewing gums | 30 |
| Supermarket brand diet fizzy drinks | 29 |
| Splenda | 16 |
| Dr Pepper | 13 |
| Morrisons Cherry /Asda/Tesco Cordials/Lucozade sugar free | 9 |
| Truvia Stevia | 9 |
| Irn Bru | 6 |
| McVities/ Maryland sugar free biscuits | 3 |
| Cadbury/Galaxy light instant hot chocolate | 2 |
| Alpen light bars | 1 |
| Activia diet yogurt | 1 |
| M&S low calorie coleslaw | 1 |
| Tesco custard No added sugar | 1 |
