# Supplementary material for: Knowledge and Perceptions of Non-Nutritive Sweeteners Within the UK Adult Population
Source: Nutrients. 2021 Jan 29;13(2):444. doi: 10.3390/nu13020444 (PMC7911612; doi:10.3390/nu13020444)
Supplement: Supplementary file 1 [file nutrients-13-00444-s001.zip › suppl/Survey.pdf]

Consumers’ knowledge and safety perceptions of artificial sweeteners

Information for participants

Thank you for your interest in taking part in this survey. Please find below further information about this study that should help you decide whether you would like to take part.

Background

Artificial sweeteners, also commonly known as **non-nutritive sweeteners**, are substances used instead of sugar to sweeten foods, beverages and other products. They provide very little or no calories. You may also know them as **low-calorie sweeteners**, or **non-caloric sweeteners.**

What will my participation involve?

You will be invited to participate in an online questionnaire to evaluate your knowledge and perceptions of artificial sweeteners, and their associated benefits, safety and risks. The survey is expected to take **no more than 10 minutes of your time.** We will then share with you some information about the regulations surrounding the use of artifical sweeteners and you will be asked to respond to a few additional questions. You will also be asked a few questions about yourself, including body perception and disease history.

Will there be any benefits to me in taking part?

You will get some information about the UK regulations governing the use of artificial sweeteners. You will also be able to contact the researcher for a report of the main findings of this research.

Will there be any risks to me in taking part?

There are no known risks associated in taking part in this study.

What happens if I decide to withdraw from the survey?

You can withdraw from completing or submitting this survey at any time by closing the page. Your participation is anonymous, and by submitting your survey, you will be consenting to the use of this information.

How will you ensure that my contribution is anonymous?

You won't be asked to share any unique traceable information (e.g date of birth, email etc.). In addition, all data collected will be kept in online software with a protected password that is only accessible to the researcher(s). Data will be transferred to a password-protected computer at the end of the survey and deleted once the project is complete.

Consent to take part in this survey

I confirm that I have read and understood the information related to this study ✱ *Required*

- ○ Yes

I understand that my participation is voluntary and that I am free to withdraw from completeing or submitting the survey by closing the page at any point during the survey. ✱ *Required*

- ○ Yes

I agree to take part in this survey and for the anonymised data to be used as the researcher sees fit, including publication. ✱ *Required*

- ○ Yes

I confirm that I am a UK resident over 18 years of age. ✱ *Required*

- ○ Yes
- ○ No

About you

Age range: ✱ *Required*

- [ ] 18 - 24
- [ ] 25 - 34
- [ ] 35 - 44
- [ ] 45 - 54
- [ ] 55 - 64
- [ ] 65+

Gender: ✱ *Required*

- [ ] Male
- [ ] Female
- [ ] Transgender

Ethnicity: ✱ *Required*

- ○ White British
- ○ White Irish
- ○ Other White
- ○ Chinese
- ○ Indian
- ○ Pakistani
- ○ Bangladeshi
- ○ Other South Asian
- ○ Black Caribbean
- ○ Black African

- Black Other
- Mixed Race
- Other

If you selected Other, please specify:

Education: ✱ *Required*

- No formal qualifications
- GCSE/ O-Level
- A-Level or Equivalent
- Degree Level
- Postgraduate Qualification
- Other

If you selected Other, please specify:

Main occupation/profession: ✱ *Required*

- Health-related Professions
- Managers, Directors and Senior Officials
- Professional Occupations (other than Health-related)
- Associate Professional and Technical Occupations
- Administrative and Secretarial Occupations

- ○ Skilled Trade Occupations
- ○ Caring, Leisure and Other Service Occupations
- ○ Sales and Customer Service Occupations
- ○ Process, Plant and Machine Operatives
- ○ Elementary Occupations
- ○ Student/ Unemployed/ Retired
- ○ Other

If you selected 'Other', please specify:

____

If you selected 'Health-related Professions', please include details about your profession:

____

If you selected 'Student', please specify your field of study:

____

Location ✱ *Required*

- ○ England
- ○ Scotland
- ○ Wales
- ○ Northern Ireland

Body perception

Do you consider yourself ✱ *Required*

- Underweight
- Normal weight
- Overweight
- Obese
- I don't know
- Prefer not to say

Are you currently on, or have ever been on, a weight loss diet? ✱ *Required*

- Yes
- No

Disease history

Do you have OR have you had any of these diseases: ✱ *Required*

- ☐ Type 1 Diabetes
- ☐ Type 2 Diabetes
- ☐ High blood pressure
- ☐ Heart disease
- ☐ Cancer
- ☐ No, I don't/haven't had any of these

Consumption of artificial sweeteners

Do you consume artificial sweeteners? ✱ *Required*

- No - not at all
- Yes

Usual consumption of artificial sweeteners

In which foods or drinks do you think you usually consume them?   (Tick as many answers as apply) ✱ *Required*

Please select at least 1 answer(s).

- ☐ In soft/fizzy drinks
- ☐ I add them to coffee and other hot beverages
- ☐ Sweets, cakes and desserts
- ☐ Chewing gums
- ☐ Other foods and drinks

If you selected 'Other food and drinks', please specify:

Please add details of any food and drink product brands with artificial sweeteners that you consume: ✱ *Required*

You consume artificial sweeteners because they: ✱ *Required*

	Strongly disagree	Disagree	Neither disagree nor agree	Agree	Strongly agree
Are tasty	☐	☐	☐	☐	☐

Are healthier than sugars	☐	☐	☐	☐	☐
Are low in calories	☐	☐	☐	☐	☐
Satisfy your sweet cravings	☐	☐	☐	☐	☐
Are ingredients in food and drink products that you consume	☐	☐	☐	☐	☐

Artificial sweeteners: acceptance, benefit and risk perceptions

How strongly you agree/disagree with the following statements? ✱ *Required*

	Strongly disagree	Disagree	Neither disagree nor agree	Agree	Strongly agree	I don't know
I don’t consume foods and drinks that contain artificial sweeteners	☐	☐	☐	☐	☐	☐
I check the labels on food & drink packaging for artificial sweeteners	☐	☐	☐	☐	☐	☐
I think that artificial sweeteners are not natural and therefore harmful	☐	☐	☐	☐	☐	☐
I think calling them "artificial" makes me sceptical about their safety	☐	☐	☐	☐	☐	☐
I think that artificial sweeteners are bad for health	☐	☐	☐	☐	☐	☐

I worry about the effects artificial sweeteners could have on my body	☐	☐	☐	☐	☐	☐
I have concerns about artificial sweeteners and the risk of cancer	☐	☐	☐	☐	☐	☐
I think artificial sweeteners can cause people to gain weight	☐	☐	☐	☐	☐	☐
I think artificial sweeteners can cause diabetes	☐	☐	☐	☐	☐	☐
Artificial sweeteners can cause allergic reactions	☐	☐	☐	☐	☐	☐
Pregnant women should not consume artificial sweeteners	☐	☐	☐	☐	☐	☐

How strongly you agree/disagree with the following statements? ✱ *Required*

	Strongly disagree	Disagree	Neither disagree nor agree	Agree	Strongly agree	I don't know

I am fine with foods and drinks containing artificial sweeteners	☐	☐	☐	☐	☐	☐
I think that artificial sweeteners are absolutely safe for health	☐	☐	☐	☐	☐	☐
Artificial sweeteners are helpful for someone who wishes to lose weight	☐	☐	☐	☐	☐	☐
Artificial sweeteners are helpful for someone who has diabetes	☐	☐	☐	☐	☐	☐
Artificial sweeteners bring more benefit than risks to consumers	☐	☐	☐	☐	☐	☐
I find artificial sweeteners to benefit me personally	☐	☐	☐	☐	☐	☐
Artificial sweeteners allow me to control and reduce calories in my diet	☐	☐	☐	☐	☐	☐

Artificial sweeteners allow for a little indulgence without feelings of guilt	☐	☐	☐	☐	☐	☐
Artificial sweeteners allow for diet products to be a viable option for those looking to lose weight and/or control their sugar intake	☐	☐	☐	☐	☐	☐

Common artificial sweeteners include: aspartame, saccharin, sucralose, acesulfame K, stevia". Do you view them all in a similar way with regards to safety? If not, which one(s) do you consider differently, and why? ✱ *Required*

Approval and Regulation

There are regulations in place governing the types of artifical sweeteners that can be added to food and drink products (as well as the amounts of particular artificial sweeteners that can added to specific products). It is the European Food Safety Authority (EFSA) which approves the use of sweeteners in foods and drinks, and the Food Standards Agency (FSA) that has responsibility for enforcing regulations in the UK.

Where do you get information relating to the benefits and safety of artificial sweeteners? (Tick as many answers as apply). ✱ *Required*

- ☐ TV/radio
- ☐ Twitter
- ☐ Instagram
- ☐ Facebook
- ☐ Scientific reports and research papers
- ☐ Government health agencies websites - e.g. NHS
- ☐ Government food regulator's website - e.g. Food Standards Agency
- ☐ Health and wellness blogs or websites
- ☐ Other

If you selected Other, please specify:

How strongly do you agree/disagree with these statements? ✱ *Required*

	Strongly disagree	Disagree	Neither disagree nor agree	Agree	Strongly agree	I don't know
I trust information coming from Government health agencies (such as the NHS)	☐	☐	☐	☐	☐	☐
I trust information coming from food regulatory authorities (such as EFSA or FSA)	☐	☐	☐	☐	☐	☐
I trust information coming from research/scientific papers	☐	☐	☐	☐	☐	☐
I trust information coming from health and wellness blogs	☐	☐	☐	☐	☐	☐
I trust information coming from social media	☐	☐	☐	☐	☐	☐

Regulation of artificial sweeteners ✱ *Required*

	Strongly disagree	Disagree	Neither disagree nor agree	Agree	Strongly agree	I don't know

I am aware of the regulations surrounding the use of artificial sweeteners	☐	☐	☐	☐	☐	☐
I am not aware of these regulations as I don't know where to look for them	☐	☐	☐	☐	☐	☐
I am not aware of these regulations as I am not motivated enough to look for them	☐	☐	☐	☐	☐	☐
I trust the regulatory bodies as their aim is to protect consumers' health	☐	☐	☐	☐	☐	☐
I trust the regulators's position (such as EFSA and FSA) regarding the safety and benefits of artificial sweeteners	☐	☐	☐	☐	☐	☐

Regulations mean that only a safe amount of these sweeteners are available in food and drinks	☐	☐	☐	☐	☐	☐
All artificial sweeteners have been vigorously tested before being allowed on to the market	☐	☐	☐	☐	☐	☐

Position of professional and regulatory bodies in relation to the use of artificial sweeteners

The BDA (British Dietetic Association) is a professional association representing dietitians in the UK, and one of the oldest and most experienced dietetic organisations in the world. Here is what BDA says about the use of artifical sweeteners:

1. Artificial sweeteners available to purchase in the UK are safe for consumption in adults up to the acceptable daily intake, and are authorised and approved for use by EFSA (European Food Safety Authority).

Please note the Acceptable Daily Intake is very high and it is unlikely that the diet of any individual will provide this amount.

2. To date, there is substantial evidence to suggest that artificial sweeteners do NOT cause cancer, lymphoma, leukaemia, chronic fatigue syndrome, Parkinson's disease, Alzheimer's disease, multiple sclerosis, autism or lupus.

3. Artificial sweeteners can help in the management of weight and other health conditions such as diabetes. A high sugar/fat diet can cause weight gain and other conditions such as Type 2 diabetes and heart disease. Therefore, artificial sweeteners might help control calories and sugar intake.

"EFSA provides scientific analysis, opinion and recommendations to support policy development on food safety issues in the European Union (EU). All artificial sweeteners used in the EU have undergone safety evaluation (e.g., toxicological testing) before being approved for use. The Joint Food and Agriculture Organisation (FAO)/World Health Organisation (WHO) Expert Committee on Food Additives (JECFA) and UK Government regulate and authorise the use of artificial sweeteners, providing food manufacturers with stringent guidelines on the maximum quantity of artificial sweetener that can be added to foods and drinks".

Post-survey knowledge

Were you previously aware of this information? ✱ *Required*

- ◯ Yes
- ◯ Mostly yes
- ◯ No, I wasn't aware of this information

Had you known this information before, would it have helped you be less concerned about the use of artificial sweeteners? ✱ *Required*

- ◯ Yes
- ◯ Maybe
- ◯ No, I would still be concerned
- ◯ No, I am already not concerned
- ◯ Other

If you selected 'Other', please add more details here:

How would you want to see such information communicated to you? (Tick as many as apply) ✱ *Required*

- ☐ Media (TV/radio)
- ☐ Internet
- ☐ Leaflets and posters

- [ ] Food labels on food & drink products
- [ ] Other

If you selected Other, please specify:

Have you changed any of your opinions in relation to artificial sweeteners after reading this information? ✱ *Required*

More info

- ( ) Yes
- ( ) Maybe
- ( ) No, this was already my position
- ( ) No, I am still not convinced

Perceptions of artificial sweeteners

How strongly do you now agree/disagree with the following statements? ✱ *Required*

	Strongly disagree	Disagree	Neither disagree nor agree	Agree	Strongly agree	I don't know
I think that artificial sweeteners are bad for health	☐	☐	☐	☐	☐	☐
I worry about the effects artificial sweeteners could have on my body	☐	☐	☐	☐	☐	☐
I have concerns about artificial sweeteners and the risk of cancer	☐	☐	☐	☐	☐	☐
I think artificial sweeteners can cause people to gain weight	☐	☐	☐	☐	☐	☐
I think artificial sweeteners can cause diabetes	☐	☐	☐	☐	☐	☐
I think that artificial sweeteners are completely safe for health	☐	☐	☐	☐	☐	☐

Artificial sweeteners are helpful for someone who wishes to lose weight	☐	☐	☐	☐	☐	☐
Artificial sweeteners are helpful for someone who has diabetes	☐	☐	☐	☐	☐	☐
Artificial sweeteners have more benefits than risks for consumers	☐	☐	☐	☐	☐	☐

End of survey

Please click on Finish to complete the survey

Thank you

Survey completed.

Many thanks for taking part!
